# Steam Explosion Enhances the Powder Property, Instant Solubility, and Diffusivity of Superfine Ground Tea Powder

**DOI:** 10.3390/foods14081345

**Published:** 2025-04-14

**Authors:** Xin Zhuang, Yue Chen, Shuiqing Wang, Kai Zhong, Wenjie Sui, Chao Ma, Maoyu Wu

**Affiliations:** 1 State Key Laboratory of Food Nutrition and Safety, College of Food Science and Engineering, Tianjin University of Science & Technology, Tianjin 300457, China; 22842074@mail.tust.edu.cn (X.Z.); 22844948@mail.tust.edu.cn (Y.C.); 22844949@mail.tust.edu.cn (S.W.); z15079588983@163.com (K.Z.); 2 Jinan Fruit Research Institute, All-China Federation of Supply & Marketing Co-Operatives, Jinan 250014, China; mch.01@163.com

**Keywords:** low-grade tea, steam explosion, superfine grinding, extraction kinetics, powder property

## Abstract

Low-grade tea, often underutilized due to its coarse texture and limited bioavailability, represents a significant resource waste. This study systematically investigated the synergistic effects of steam explosion (SE) and superfine grinding on enhancing the structural deconstruction, powder property, instant solubility, and diffusivity of low-grade. SE treatment induced critical physicochemical modifications, including hemicellulose degradation, lignin recondensation, and cellulose crystalline reorganization, which significantly weakened the lignocellulosic matrix. Subsequent superfine grinding via ball milling achieved ultrafine particles, with median diameter *D*_50_ = 10.4 ± 0.17 μm, and almost completely destroyed the cell wall by 99.9%. Extraction kinetics revealed that SE-ball milling synergistically accelerated the diffusion behavior of bioactive compounds, reducing equilibrium time by 2~4 times and increasing maximum yields of polysaccharides, polyphenols, caffeine, and water-soluble solids by 9~25% compared to untreated samples. Homogenization combined with 0.08 mg/mL CMC-Na further improved the suspension stability of tea powder and reduced its centrifugal sedimentation to 9.85%. These findings demonstrate a scalable strategy to transform low-grade tea into high-value ingredients with enhanced accessibility and solubility of bioactive compounds, offering promising applications in instant beverages, fortified foods, and nutraceuticals.

## 1. Introduction

Tea is one of the most widely consumed beverages globally, attracting significant attention due to its bioactive compounds [1,2]. Among these, polyphenols exhibit strong antioxidant properties, neutralizing free radicals and reducing the risk of inflammation and cardiovascular diseases [3]. Caffeine stimulates the central nervous system, enhancing alertness and metabolic rate. Polysaccharides contribute to immune function, regulate blood sugar levels, and improve gut health [4]. Collectively, these compounds interact synergistically to not only enhance the sensory experience of tea but also significantly bolster its health benefits. However, the industry predominantly focuses on high-grade leaves, while low-grade tea (including coarse leaves, stems, and processing residues) faces an annual surplus or backlog of approximately 100,000 tons in China, is often discarded or relegated to low-value applications due to its poor solubility, high fiber content, and limited bioavailability of active compounds. Conventional methods like mechanical grinding or enzymatic hydrolysis inadequately address these challenges, yielding coarse particles or requiring stringent process control [5]. Recent advancements in biomass valorization highlight steam explosion (SE) as a promising technique for lignocellulosic deconstruction [6], yet its integration with superfine grinding for tea processing, which is expected to produce diversified products such as instant tea powders and tea extracts, remains underexplored. This study hypothesizes that SE-induced structural weakening, combined with mechanical refinement, can overcome bioavailability barriers in low-grade tea. We systematically evaluate the physicochemical modifications, particle properties, and extraction kinetics of SE-treated tea powder, aiming to establish an efficient pathway for industrial-scale production of functional tea ingredients.

Conventional approaches to upgrading the utilization of low-grade tea, such as mechanical grinding, microbial fermentation, and enzymatic hydrolysis, aim to enhance solubility and bioactive compound accessibility [7]. While these methods partially improve physicochemical properties, they face inherent limitations. For example, mechanical grinding produces coarse particles with incomplete cell wall disruption, restricting the release of intracellular components. Enzymatic hydrolysis, although effective in degrading lignocellulosic structures, tends to require longer processing times, and the enzymatic process is affected by various factors, such as temperature, pH, and time, which need to be precisely controlled. Similarly, microbial fermentation may suffer from flavor instability or short shelf life, requiring additional preservation processing measures. These challenges highlight the need for innovative technologies that can effectively modify the tea matrix while maintaining bioactive integrity.

SE is recognized as one of the most effective plant cell wall deconstruction techniques [8]. SE induces physicochemical modifications through a two-phase mechanism: (1) high-temperature steam hydrolysis softens lignocellulosic structures by hydrolyzing hemicellulose and lignin-carbohydrate complexes, and (2) instantaneous pressure release physically disrupts the plant matrix via explosive decompression [8,9]. This dual action creates microcracks and porous architectures, unclogging bioactive compound transfer pathways [10]. For example, plant fatty acids, phytosterols and volatile compounds were more easily extracted by solvents after SE treatment. The extraction of curcumin from turmeric after SE treatment was 3.24% (*w*/*w*), which is comparable to the extraction of 3.98% (*w*/*w*) from finely ground turmeric [9].

Superfine grinding technology has been widely used in the food industry, especially in the value-added utilization of food processing by-products. During food processing, by-products such as fruit and seed peels and tea waste often retain important nutritional value but are discarded due to their coarse texture and poor solubility. Superfine grinding addresses these limitations by reducing particle size to the micron or submicron level, thereby increasing surface area and disrupting cell wall structure [10]. Similarly, cereal bran, traditionally considered a low-value by-product of grinding, has been superfine ground to improve its water-binding capacity and antioxidant activity, allowing it to be added to baked goods and extruded snacks [11,12]. Superfine grinding of residues from soybean processing treatments has been performed and has resulted in a significant increase in physicochemical properties and antioxidant activity [13]. However, standalone superfine grinding faces challenges, including particle aggregation, oxidative instability, and high energy consumption, particularly for fibrous materials like low-grade tea. Here, integrating SE pretreatment offers a synergistic solution: SE weakens the lignocellulosic matrix, reducing grinding energy demands while enabling finer particle sizes and higher cell wall breakage ratios. For example, compared to untreated samples, bran powder treated with steam explosion-assisted ultrafine grinding (SESG) had a lower particle size (<75 μm) and a higher water solubility index (211.75 mg/g) [14].

Complementary to SE, cryogenic grinding and ball milling are widely adopted for superfine powder production. Cryogenic grinding minimizes the thermal degradation of heat-sensitive bioactive substances (such as polyphenols) by maintaining subzero temperatures during pulverization [15]. Ball milling employs mechanical impact and shear forces to achieve micron-scale particles, enhancing dispersibility and bioavailability [16]. While both methods improve functional properties, their efficiency is limited when applied to intact plant tissues with robust cell walls. This limitation is overcome by SE pretreatment, which creates structural defects (such as microfractures and pore networks), enabling mechanical grinders to target weakened regions and achieve uniform particle distributions with reduced processing time. The synergistic potential of SE and superfine grinding for low-grade tea processing remains underexplored. SE materials often exhibit irregular morphology and residual coarse particles, necessitating further refinement to meet industrial standards for powder additives. Superfine grinding not only enhances particle uniformity but also improves aesthetic and functional properties, such as flowability and suspension stability, which are critical for applications in instant beverages and fortified foods [17]. Moreover, SE-induced porosity facilitates solvent penetration during extraction, while ultrafine particles maximize bioactive compound release kinetics’ dual advantage, yet to be systematically quantified for tea systems.

This study innovatively combined SE with superfine grinding to process low-grade tea. The research systematically investigates the synergistic effects of SE and superfine grinding on the microstructure, particle size distribution, and cell wall breakage ratio of tea powder, aiming to optimize settling performance and dispersion stability. The objectives of this study were to establish a scalable and efficient pathway for producing high-quality tea powder. The findings are expected to advance industrial applications in functional foods, instant beverages, and nutraceuticals while providing theoretical insights into the interplay between bioactive compound retention, structural alterations, and nutritional functionality.

## 2. Materials and Methods

### 2.1. Materials

Low-grade tea was purchased from Yichang Wufeng Tea Factory, Yichang City, Hubei Province, China. Four stabilizing agents, sodium carboxymethyl cellulose (CMC-Na), sodium alginate, agar, and xanthan gum, were purchased from Sinopharm Group Chemical Reagent Co., Ltd. (Tianjin, China, analytical grade). The gallic acid standard (CAS: 149-91-7, >98%), tea polyphenol standard (CAS: 84650-60-2, >98%), and caffeine standard (CAS: 58-08-2, >99.8%) were purchased from Beijing Dingguo Changsheng Biotechnology Co., Ltd. (Beijing, China). Methanol (CAS: 67-56-1, chromatographic grade, 99.9%) was purchased from Tianjin Jiangtian Chemical Technology Co., Ltd. (Tianjin, China); formic acid (CAS: 64-18-6, chromatographic grade, 99.8%) was obtained from Tianjin Comio Chemical Reagent Co., Ltd. (Tianjin, China); ethanol (CAS: 64-17-5, analytical grade, 99.99%), sodium chloride (CAS: 7647-14-5, analytical grade, 99.8%), glacial acetic acid (CAS: A116166, analytical grade, 99.5%), and hydrochloric acid (CAS: 1185-53-1, analytical grade) was supplied by Tianjin Chemical Reagent No. 1 Factory (Tianjin, China).

### 2.2. Steam Explosion Process

SE was performed using a lab-made batch SE apparatus with a vessel capacity of 5 L. Before steam explosion treatment: 300 g of low-grade tea was uniformly moisturized to 30% water content (*w*/*w*) and subsequently loaded into the reaction vessel. The system pressure and temperature were progressively elevated through saturated steam injection, with sustained maintenance at five pressure levels: 0.2 MPa, 0.4 MPa, 0.6 MPa, 0.8 MPa, and 1.0 MPa, each for 3 min. The corresponding SE severities were determined as 1.07, 1.76, 2.21, 2.55, and 2.83 under these respective pressure conditions. After opening the ball valve to achieve a rapid decompression, the exploded samples were recovered into the receiver. SE severity factor (log*R*_0_) can be calculated from the following equation [18]:

(1) $$R_{0}=t_{r}exp\left(\frac{T-100}{14.75}\right)$$

where *t_r_* is the retention time (min) and *T* is the holding temperature (°C).

In order to investigate the energy consumption characteristics of SE in industrial production to advance its application, the total energy consumption (*Q_t_*) and unit dry matter steam consumption (*m^′^*) of the SE process were calculated in this study [19]:

(2) $$Q_{t}=m_{g}\left(h_{ge}-h_{f0}\right)+\left[C_{v}m_{a}+\left(C_{p}m\left(1-w\right)+C_{w}mw\right)+C_{r}M_{r}\right]\left(T_{e}-T_{0}\right)+\varepsilon C_{0}T_{e}^{4}A_{r}t\times 10^{-5}$$

where $m_{g}=\frac{P_{0}V}{RT_{0}}$ is the amount of steam in reactor (kg) (*P*_0_ is the initial surrounding pressure (Pa), *V* is the volume of the reactor (m^3^), *R* is the gas constant, *T*_0_ is the initial surrounding temperature (K)); *h_ge_* is the enthalpy of steam at *T_e_* (kJ/kg); *h_f_*_0_: is the enthalpy of water at *T*_0_ (kJ/kg); *C_v_* is the specific heat capacities of air at constant volume (kJ/kg·K); *m_a_* is the amount of air in reactor (kg); *C_p_*, *C_w_* is the specific heat capacities of dry matter/water (kJ/kg·K); *m* is the amount of material (kg); *w* is the moisture content of low-grade tea (wt%); *C_r_* is the specific heat capacities of reactor’s material(kJ/kg·K); *M_r_* is the weight of reactor (kg); *T_e_* is the holding temperature (K); *ε* is the blackness of reactor’s material; *C*_0_ is the blackbody radiation coefficient (W/(m^2^·K^4^)); *A_r_* is the heating area of reactor (m^2^); *t* is the retention time (min).

(3) $$m^{\prime }=\frac{Q_{t}}{\left(h_{ge}-h_{fo}\right)\times m\left(1-w\right)}\;\;$$

To optimize industrial scalability, a multivariate regression model was developed:

$$m’=0.77+0.051B+0.38C-0.48D+1.28E-0.034BD-0.13CD+0.18CE+0.57D^{2}+0.82E^{2}$$

where: *B* is the loading factor (kg/m^3^); *C* is the moisture content of low-grade tea (wt%); *D* is the holding temperature (K); *E* is the retention time (min).

### 2.3. Determination of Physicochemical Properties of Low-Grade Tea

#### 2.3.1. Chemical Composition Determination

The basic chemical constituents of low-grade tea, including alcohol extractives, cellulose, hemicellulose, acid-insoluble lignin (AIL), and acid-soluble lignin (ASL), were determined according to the laboratory analytical procedures of the National Renewable Energy Laboratory (NREL). The specific methods were as follows: Water extracts were determined using deionized water as the solvent, continuously extracted in a Soxhlet extractor for more than 6 h; ethanol extracts utilized anhydrous ethanol as the solvent, refluxing in a Soxhlet extractor for more than 6 h. The remaining solid residue was accurately weighed, and 3 mL of sulfuric acid and 72 mL of deionized water were added. The mixture was then treated in an autoclave for 1 h. Subsequently, approximately 50 mL of the aliquot was transferred to a sample storage bottle. This sample is suitable for the determination of acid-soluble lignin and carbohydrates, and if necessary, the acetyl content was also analyzed. The chromatographic conditions were set as follows: the acetyl CTO-20A liquid chromatography equipment (Shimadzu, Kyoto, Japan) with an Aminex HPX-87H column (300 × 7.8 mm, polystyrene-divinylbenzene resin) was employed. The mobile phase consisted of 0.005 mol/L H_2_SO_4_ solution delivered at a flow rate of 0.6 mL/min. The separation was carried out at a column temperature of 65 °C with an injection volume of 20 μL. Detection was performed using a refractive index detector (RID). Acid-soluble lignin was measured using ultraviolet spectrophotometry, determining the absorbance value at a wavelength of 240 nm. To ensure the accuracy and reliability of the results, the measurement must be completed within 6 h after hydrolysis. For acid-insoluble lignin, the crucible and acid-insoluble residue were dried in a constant temperature oven at 105 ± 3 °C until a constant weight was achieved. This process typically requires at least 4 h to thoroughly remove moisture interference, ensuring the precision of the measurement data [20].

#### 2.3.2. Crystallinity Degree Determination

The low-grade tea was ground into powder, sieved through a 100-mesh sieve, and subsequently dried. An AD8 Advance A25 X-ray diffractometer (Bruker, Ettlingen, Germany) was used to measure the crystallinity degree of low-grade tea with Cu Kα (*λ* = 1.5406 Å) radiation source at an angle of 5–65° and a speed of 2°/min. The crystallinity degree was calculated from the following equation:

(4) $$\mathrm{Crystallinity}\%=\left(I_{002}-I_{am}\right)/I_{002}\times 100\%$$

where *I*_002_ represents the intensity of the (002) diffraction peak at approximately 2*θ* = 22.5°, and *I_am_* represents the background intensity at approximately 2*θ* = 18.3°.

#### 2.3.3. Thermal Stability Determination

The denaturation temperature of the samples was determined using a differential scanning calorimeter equipped with a TA-60WS detection system (Shimadzu, Kyoto, Japan), based on the analysis of DSC curves. Approximately 2 mg of the sample was placed in an aluminum crucible for the DSC and heated from 30 °C to 598 °C at a rate of 10 °C/min [21].

### 2.4. Preparation of Superfine Tea Powder

Low-grade tea was uniformly rehydrated to 30% moisture content and processed using two distinct methodologies. For cryogenic grinding, untreated tea was directly pulverized in a cryogenic grinder (DHC-250, Shanghai Heaven Precision Instrument Co., Ltd., Shanghai, China) to produce raw cryogenically ground tea powder (R-TP_C_). SE pretreatment was alternatively applied by subjecting tea to saturated steam at 170.44 °C for 3 min, followed by instantaneous pressure release; the SE-treated tea was then cryogenically ground under identical conditions to yield steam-exploded cryogenically ground tea powder (SE-TP_C_). For ball milling, untreated tea was mixed with zirconium oxide grinding media at a 1:2 (*v*/*v*) ratio and processed in a planetary ball mill (CJM-SY-B TJ-0.4L, Tianjin Oriental Tianjing Technology Development Co., Tianjing, China) at 400 rpm for 4 h to obtain raw ball-milled tea powder (R-TP_B4_) [22,23]. Steam-exploded counterparts were prepared by subjecting tea to identical SE pretreatment before ball milling under the same operational parameters, producing steam-exploded ball-milled tea powder (SE-TP_B4_). The final product was dried at 45 °C for 48 h, stored in polyethylene bags, and preserved at 4 °C before further analysis.

### 2.5. Microscopy Observation of Superfine Tea Powder

The tea powder samples were observed using a JEOL JSM-3800F system (JEOL, Kyoto, Japan) to obtain SEM images at ×100 and ×200 magnifications. Before measurement, the samples were dried in an oven at 60 °C. Then, they were coated with a thin layer of gold using a sputter-coater (Hitachi Science Systems, Kyoto, Japan). Scanning electron microscopy (SEM) analysis was conducted using a secondary electron detector with a resolution of 30 nm (at 30 kV), an accelerating voltage range of 0.3 to 30 kV, and a chamber vacuum pressure of 1.5 × 10^−3^ Pa.

### 2.6. Particle Size Distribution and Cell Wall Breakage Ratio of Superfine Tea Powder

The particle size distribution of the tea powder (0.1–716 μm) was measured using a BT-9300S laser diffraction particle size analyzer (Beckman Coulter, Brea, CA, USA) with water as the solvent at room temperature. The particle size characteristics were expressed as the median diameter (*D*_50_) and span factor [(*D*_90_ − *D*_10_)/*D*_50_], where *D*_10_, *D*_50_, and *D*_90_ represent the particle size at 10%, 50%, and 90% cumulative percentages, respectively. The sample (refractive index = 1.54) in an aqueous dispersion medium (refractive index = 1.33) was analyzed using a laser wavelength of 650 nm. Prior to analysis, the particles were pre-treated with 50 W ultrasonic dispersion for 1 min, followed by homogenization at a pump speed of 5000 rpm to ensure uniform suspension [24].

The typical cell diameter of plant materials is generally considered to range from 10 to 20 μm. When the median particle size was *D*_50_ > 10 μm, the cell wall breakage ratio was calculated using the following equation [25]:

(5) $$U=1-{\left(1-10/D_{50}\right)}^{3}$$

When the median particle size was *D*_50_ < 10 μm, *U* = 100%.

### 2.7. Sedimentation Performance and Dispersion Optimization of Superfine Tea Powder

The cryogenically ground and ball-milled raw materials, along with SE tea powder, were dispersed in deionized water at a ratio of 1:1000 (*w*/*w*), followed by 10 min stirring to form a 0.1% (*w*/*v*) tea-water mixture. Four stabilizers (CMC-Na, sodium alginate, agar, and xanthan gum) were individually incorporated into the 0.1% tea-water mixture at concentrations ranging from 0.08 to 0.24 mg/mL. The mixtures were homogenized using an ATS GL-20G-C high-pressure shear homogenizer at 500 bar. After homogenization, samples were collected and allowed to stand for 2 h. The gravimetric sedimentation rate was determined through static settling, while the centrifugal sedimentation rate was measured by centrifugation at 4000 rpm for 10 min [23]. The calculation formula for the gravitational and centrifugal sedimentation ratio:

(6) $$\mathrm{Gravitational}\;\mathrm{sedimentation}\;\mathrm{ratio}\%=\left(m_{1}-m_{2}\right)/m\times 100\%$$

(7) $$\mathrm{Centrifugal}\;\mathrm{sedimentation}\;\mathrm{ratio}\%=\left(m_{1}^{\prime }-m_{2}^{\prime }\right)/m^{\prime }\times 100\%$$

where *m*_1_ was the total dry weight of the centrifuge tube and sediment after 2 h of static settling; *m*_2_ was the dry weight of the empty centrifuge tube; m was the initial dry weight of the added tea powder; *m^′^*_1_ was the total dry weight of the centrifuge tube and sediment after centrifugation; *m^′^*_2_ was the dry weight of the centrifuged empty tube; *m^′^* was the initial dry weight of the tea powder for the centrifugal test.

### 2.8. Diffusion Property Rules of Bioactive Compounds of Superfine Tea Powder

The water-soluble carbohydrates, total polyphenols, and caffeine contents were determined according to the Chinese National Standard GB/T 8305. All results are expressed as mass percentages relative to the dry weight basis of the tea powder.

To study the mass transfer kinetics during the extraction of bioactive compounds from tea powder, Fick’s second law was applied to model the process. Based on the total extraction time of active substances from tea powder, seven measurement points were selected (5 min, 10 min, 15 min, 30 min, 45 min, 60 min, 90 min) for model fitting. After plotting the extraction points, the Box–Lucas model was used for nonlinear fitting to obtain the extraction curves. Extraction runs were analyzed with a mathematical model derived from Fick’s second law. The model is based on the following assumptions: (1) The height/diameter ratio of SE Radix Astragali particles is approximately 1. (2) Solvent mixing is complete. (3) The transport of active components within the particles is a diffusion phenomenon. (4) The diffusion of active components and other compounds occurs in parallel, without interactions between them. Mass transfer of the active ingredients in the solid follows:

(8) $$Y_{t}=Y_{\infty }\left(1-exp^{\left(-Kt\right)}\right)$$

where *Y_t_* was the bioactive extracts’ yield at time t, % (*w*/*w*, dry weight); *Y_∞_* was the maximum bioactive extracts’ yield when time approached infinity, % (*w*/*w*, dry weight); *K* was the specific rate of bioactive extracts yield, s^−1^; *t* was the extraction time, s. All batch experiments in this study were performed in triplicate.

### 2.9. Statistical Analysis

Unless otherwise indicated, all experiments were conducted in triplicate using independently prepared sample batches. Data are expressed as mean ± standard deviation (SD) from three replicates. The statistical analysis was performed employing SPSS software (version 16.0) with statistical significance defined as *p* < 0.05. Fresh reagent batches were utilized for each experimental repetition to ensure process independence.

## 3. Results and Discussion

### 3.1. Effects of Steam Explosion on Chemical Properties of Low-Grade Tea

#### 3.1.1. Chemical Composition

**Figure 1:**
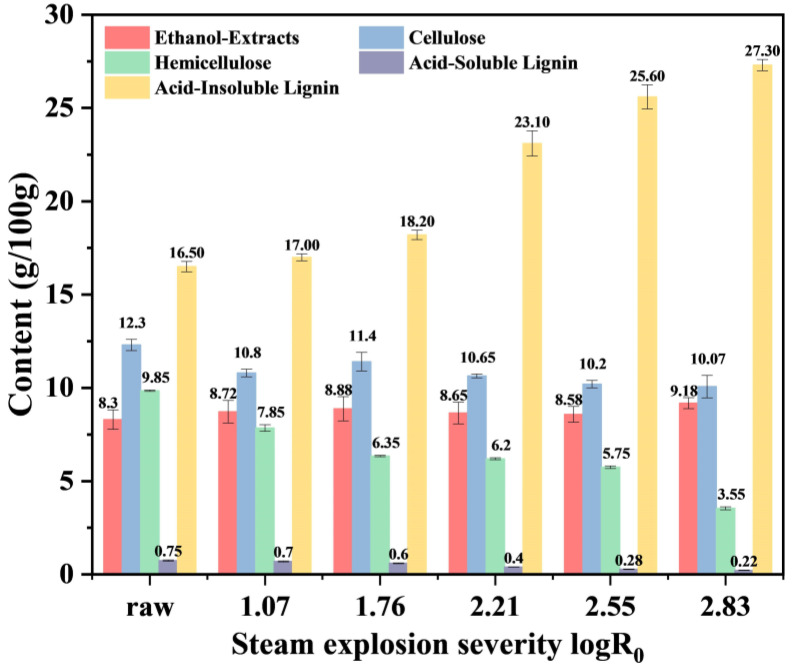
Content of low-grade tea components under different steam explosion severities.

The chemical composition of low-grade tea before and after SE treatment is shown in Figure 1. The proportion of ethanol extracts was also increased by up to 1.11-fold from 8.30 ± 0.52 wt% to 9.18 ± 0.30 wt% after SE treatment. Much of the literature has confirmed that SE could greatly promote the dissolution and release of soluble substances under certain solvents [26]. As observed, hemicellulose was dramatically reduced from 9.85 ± 0.03 wt% to only 3.55 ± 0.08 wt%; the cellulose percentage reduced from 12.30 ± 0.30 wt% to 10.07 ± 0.60 wt%; whereas the AIL percentage increased from 16.50 ± 0.28 wt% to 27.30 ± 0.30 wt% and the ASL percentage decreased from 0.75 ± 0.03 wt% to 0.22 ± 0.01 wt%.

As for cellulose, it presents a reduction tendency after SE treatment. According to previous studies, amorphous cellulose could be released and degraded into oligosaccharides or monosaccharides by SE [26]. The primary effect of SE on the chemical composition of low-grade tea is the degradation of hemicellulose. 63.96% of the hemicellulose fraction was removed under the most severe SE condition. When carried out in a hydrothermal atmosphere, hemicellulose may undergo partial autohydrolysis conversion to monosaccharides and oligomers [27]. Instantaneous exposure to high-pressure steam promoted the production of acetic acids from hemicelluloses and exacerbated the hydrolysis of hemicellulose glycosidic bonds and lignin aryl ether bonds, leading to the further degradation of monosaccharides to furfural and 5-HMF [8]. The ASL proportion of low-grade tea decreased after SE, while the AIL proportion showed an increasing trend. According to the literature, this increase can be attributed to the formation of extraneous polymeric lignin-like materials (“pseudo lignin”) by condensation reactions of carbohydrate and lignin degradation products [28].

#### 3.1.2. Crystallinity Index

**Figure 2:**
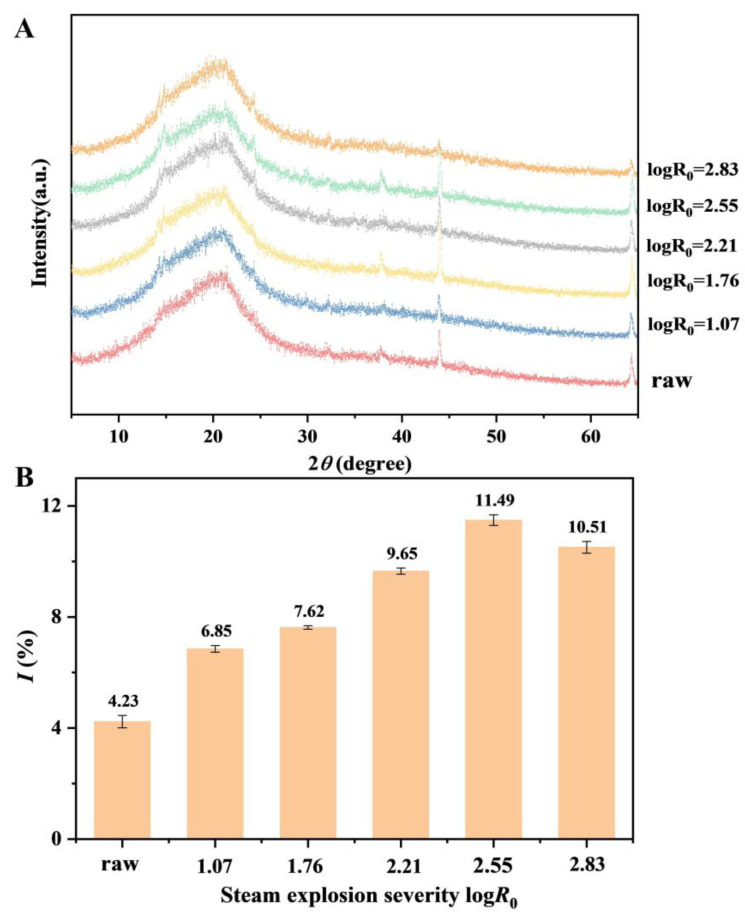
X-ray diffraction results of low-grade tea under different severities of steam explosion treatment. X-ray diffraction pattern (**A**); Crystallinity index change (**B**).

As shown in Figure 2, the majority of the peaks observed in the spectra of SE-treated samples were similar to those detected in the raw sample. This indicates that the SE treatment did not significantly disrupt the crystal structure of low-grade tea. Notably, the crystallinity index exhibited an initial rise followed by a decline as tea SE severity increased. This phenomenon may be attributed to the differential hydrolysis kinetics between cellulose and hemicellulose under hydrothermal acidic conditions. Unlike hemicellulose, cellulose comprises a biphasic system of crystalline and amorphous regions. The hydrolysis accessibility differed significantly between these regions, with the crystalline cellulose exhibiting substantially lower hydrolysis rate constants compared to the amorphous regions. The amorphous cellulose underwent rapid hydrolysis into oligosaccharides and subsequently into monosaccharides, whereas the crystalline domains demonstrated greater resistance. Therefore, it is speculated that with the increase in severity of SE, the amorphous region of cellulose was destroyed, and the content of the crystal region is relatively reduced, resulting in a change in crystal structure.

#### 3.1.3. Thermal Stability

**Figure 3:**
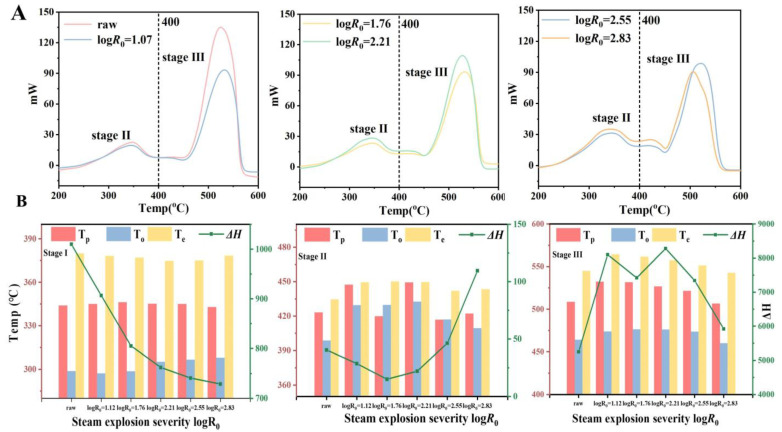
DSC spectrum of tea under different steam explosion temperatures (**A**); DSC results of low-grade tea under different steam explosion temperatures (**B**).

Figure 3A shows the thermodynamic curves of low-grade tea at various SE severities, revealing three distinct degradation stages. The first stage (30~200 °C) is mainly attributed to moisture volatilization, the second stage (200~400 °C) involves the decomposition of amorphous cellulose, hemicellulose, and oxygen-containing functional groups, and the cleavage of crystalline cellulose domains, lignin macromolecules, and sugar condensates characterizes the third stage (400~550 °C). Figure 3B shows significant changes in the thermodynamic parameters of the various stages as the severity of the steam explosion increases.

In the second stage of the pyrolysis process (200~400 °C), the hemicellulose degradation peak exhibited pronounced tailing, with Δ*H* values decreasing significantly from 1010.00 J/g to 529.53 J/g. Glycosidic bond cleavage dominated within this temperature range, generating gaseous products (such as CO_2_ and CH_4_) and oxygenated organics. The disruption of amorphous regions by SE accelerated this degradation pathway, while residual salts and carbonized byproducts likely contributed to peak broadening mechanisms. Notably, cellulose displayed a non-monotonic Δ*H* trend: initial reduction from 532.17 J/g to 421.83 J/g under moderate SE, followed by partial recovery to 487.36 J/g under severe treatment. This biphasic behavior suggests structural reorganization of cellulose microfibrils: initial destruction of amorphous regions reduced thermal stability, while subsequent enrichment of crystalline domains enhanced heat resistance through hydrogen-bond reinforcement.

The third stage, pyrolysis behavior (400~550 °C), showed significant structural stability differences. The Δ*H* value of lignin increased from 6823 J/g to 8280 J/g, likely attributable to steam explosion-induced formation of thermally stable pseudo-lignin via β-5^′^ and β-β^′^ condensed linkages between phenylpropane units. Concurrently, the cellulose peak power intensity rose from 10 mW to 35 mW, reflecting elevated energy barriers for crystalline domain decomposition due to increased crystallinity ratios. These transformations align with structural analyses suggesting that steam explosion promotes both lignin condensation and cellulose lattice densification.

Collectively, the DSC analysis reveals that intensified SE severities reduce hemicellulose stability, modify cellulose’s amorphous-crystalline balance, and enhance lignin’s thermal resistance. These alterations align with the progressive degradation of hemicellulose, structural reorganization of cellulose, and condensation reactions within lignin during SE processing.

### 3.2. Powder Property and Solubilization Property of Low-Grade Tea After Steam Explosion and Superfine Grinding

#### 3.2.1. Microstructure

**Figure 4:**
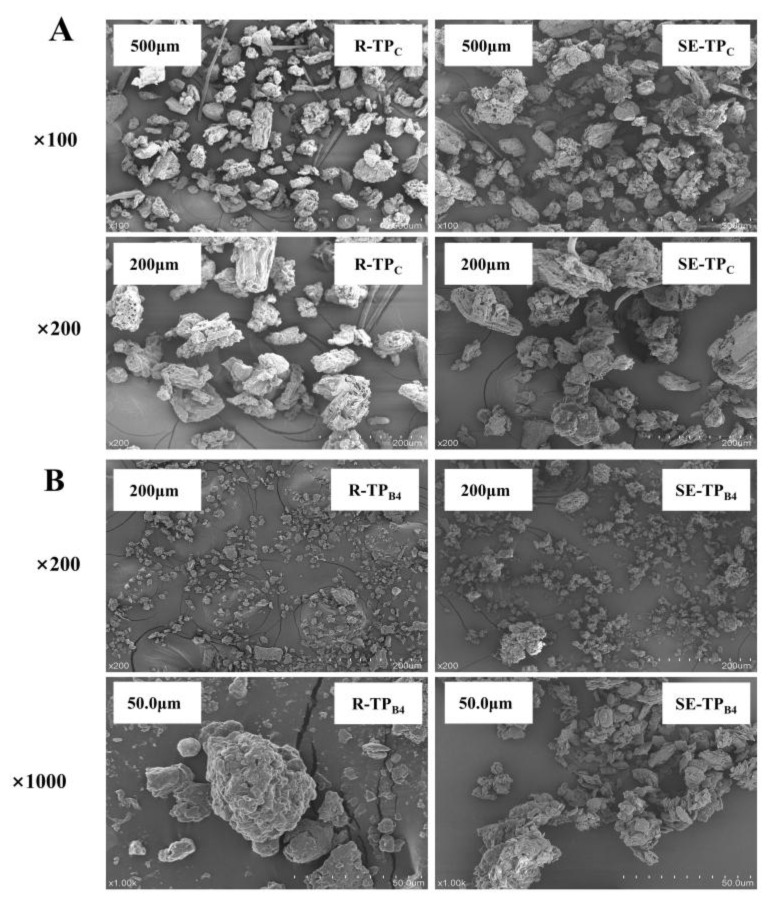
Electron microscopic images of superfine tea powder treated with different grinding conditions (**A**,**B**).

The SEM images of the four tea powders processed are presented in Figure 4. Under identical cryogenic grinding conditions, distinct morphological differences were observed between R-TP_C_ and SE-TP_C_. At ×100 magnification, R-TP_C_ exhibited incomplete pulverization characterized by the separation of veins and mesophyll alongside elliptical and irregular particles. In contrast, SE-TP_C_ displayed complete pulverization of vein structures. When magnified to ×200, R-TP_C_ retained intact vascular bundles and spiral vessel structures on particles, whereas SE-TP_C_ particles demonstrated wavy surfaces with loose porous structures. This structural alteration likely originated not only from cryogenic grinding but also from the combined effects of SE processes, including hydrothermal deconstruction and instantaneous pressure release during expansion.

Under identical ball milling conditions, both R-TP_B4_ and SE-TP_B4_ showed fragmented morphologies with uniform small particles at ×200 and ×1000 magnifications, but the SE-TP_B4_ formed finer and more homogeneous particles. Comparative analysis at ×200 magnification revealed that R-TP_B4_ particles were smaller than those of R-TP_C_, while SE-TP_B4_ displayed further reduced particle sizes and enhanced uniformity relative to SE-TP_C_. These SEM observations confirmed that SE significantly enhanced the comminution efficiency of subsequent grinding processes. The disruption of tissue structure and cellular integrity during steam explosion facilitated particle size reduction. Specifically, hydrothermal deconstruction and instantaneous pressure release phases of steam explosion may have induced microstructural damage and partial cell wall degradation, further promoting the comminution of particles during grinding.

These morphological improvements align with the chemical and structural modifications induced by SE. The partial degradation of hemicellulose and amorphous cellulose, coupled with lignin condensation reactions, likely weakened the lignocellulosic matrix, enhancing its susceptibility to mechanical fracturing. Furthermore, the increased crystallinity index under moderate SE severity suggests a denser cellulose lattice, which may have contributed to the formation of smaller, more uniform particles during grinding by concentrating stress on weakened amorphous regions. The porous architectures observed in SE-treated powders correlate with the thermal stability trends, where SE promoted lignin recondensation and cellulose crystalline structure features that facilitate crack propagation and particle disintegration under mechanical forces. Collectively, the synergy between SE-induced chemical reorganization and mechanical processing underpins the enhanced comminution efficiency and powder homogeneity.

#### 3.2.2. Particle Size Distribution and Cell Wall Breakage Ratio

**Figure 5:**
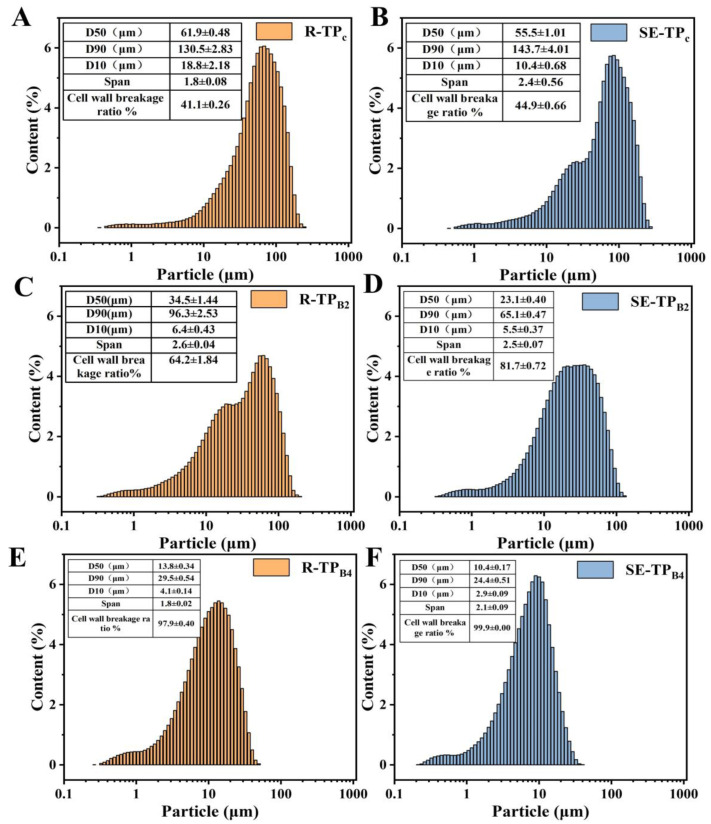
Determination of size distribution of superfine tea powder under different treatment conditions (**A**–**F**).

As illustrated in Figure 5A,B, both cryogenically ground tea powders (R-TP_C_ and SE-TP_C_) exhibited unimodal size distributions. However, SE-TP_C_ displayed an additional subsidiary peak in the smaller particle size range, indicative of more thorough fragmentation. The median particle diameter (*D*_50_) of SE-TP_C_ (54.68 μm) was significantly lower than that of untreated R-TP_C_ (60.84 μm), consistent with the SEM observations of completely pulverized leaf veins and porous structures. Following ball milling (Figure 5C–F), the refinement advantage of SE samples became more pronounced: the *D*_50_ of SE-TP_B2_ (23.1 μm) after 2 h of ball milling was 33% smaller than that of untreated R-TP_B2_ (34.5 μm). After 4 h of ball milling, SE-TP_B4_ achieved a *D*_50_ of 10.22 μm, meeting the criteria for soluble instant tea powder (*D*_50_ < 12 μm).

The SE treatment (170.44 °C, 3 min) induced dual modifications in the chemical composition and microstructure of low-grade tea through synergistic hydrothermal deconstruction and instantaneous pressure release. First, the high-temperature, high-pressure environment triggered partial hydrolysis of hemicellulose, weakening cell wall cross-linking. Second, lignin underwent acid-catalyzed condensation to form “pseudo-lignin”, which increased cell wall brittleness. These chemical alterations enhanced the susceptibility of SE-treated tea to mechanical fracturing, as evidenced by the 3.8% increase in cell wall breakage ratio for SE-TP_C_ (44.9%) compared to R-TP_C_ (41.1%). After 2 h of ball milling, SE-TP_B2_ exhibited a 17.5% higher cell wall breakage ratio (81.7%) than R-TP_B2_ (64.2%). The porous architectures observed in SE-TP_C_ further corroborated the SE-treated structural weakening.

While cryogenic grinding reduced material toughness via low-temperature embrittlement, its comminution efficiency was constrained by the inherent fibrous network of tea leaves. As shown in Figure 5, the *D*_50_ (55.5 μm) and cell wall breakage ratio (44.9%) of SE-TP_C_, though superior to those of untreated samples, remained markedly higher than those of ball-milled SE-TP_B2_ (*D*_50_ = 10.4 μm; cell wall breakage ratio = 99.9%). This disparity stems from distinct mechanical mechanisms: cryogenic grinding relies on brittle fracture, primarily disrupting macroscopic structures (such as vein separation), whereas ball milling employs dynamic impact and shearing forces from zirconium oxide balls to disintegrate microstructures (such as cell wall fragments) progressively. Prolonged ball milling (2 h to 4 h) reduced the span factor of SE-TP_B4_ from 2.5 to 2.1, reflecting enhanced particle size uniformity. Thus, ball milling demonstrates superior efficiency in ultrafine particle generation and distribution homogeneity.

In addition, the gradient change of cell wall breakage ratio (41.1% to 99.9%) further verified the decisive role of particle size refinement on the destruction of cell structure. The simultaneous decrease in *D*_50_, *D*_90_, and *D*_10_ significantly increased the specific surface area of the particles and accelerated the dissolution of cell inclusions (such as polysaccharides and polyphenols). This mechanism was particularly significant in SE-TP_B4_: the combination of its *D*_50_ = 10.22 μm and cell wall breakage ratio of 99.9% indicated that the ultrafine particles almost completely exposed the active ingredients, providing an ideal model for subsequent dispersion stability studies.

#### 3.2.3. Sedimentation Performance and Dispersion Optimization

**Figure 6:**
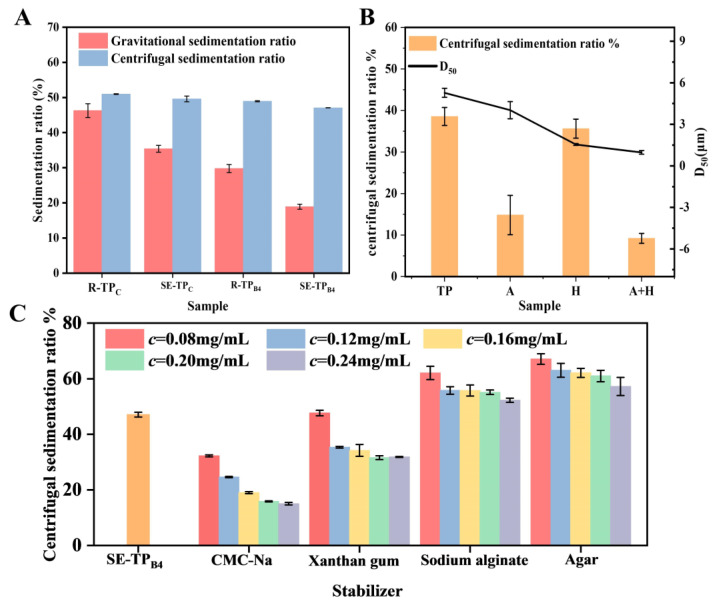
Gravity and centrifugal settlement rate of superfine tea powder under different treatment conditions (**A**); Effects of different stabilizers on centrifugal settlement rate of SE-TP_B4_ (**B**); Effects of different stabilizers on centrifugal settlement rate of SE-TP_B4_. (TP, 0.1% SE-TP_B4_ mixture (**C**). (A, 0.1% SE-TP_B4_ mixture containing 0.08 mg/mL CMC-Na; H, 0.1% SE-TP_B4_ mixture and 104 r/min homogenization; A + H, 0.1% SE-TP_B4_ mixture containing 0.08 mg/mL CMC-Na formulation and 104 r/min homogenization).

The particle size of tea powder directly influences sedimentation behavior and turbidity in beverages. As shown in Figure 6A, the gravitational and centrifugal sedimentation ratios of tea powders under different processing conditions were evaluated to determine their impact on the overall quality of the beverage. The gravitational sedimentation ratio of R-TP_C_ (46.26%) exceeded that of SE-TP_C_ by 11.15%, indicating the larger particle sizes in R-TP_C_. Following 4 h of ball milling, R-TP_B4_ exhibited a reduced gravitational sedimentation ratio of 29.77%, while SE-TP_B2_ showed a further decline to 18.92%. Centrifugal sedimentation ratios demonstrated a slight variation, with SE-TP_C_ marginally lower than R-TP_C_ and SE-TP_B4_ decreasing by 1.87% compared to R-TP_B4_. Notably, aqueous solutions of SE-TP_C_ displayed distinct stratification after 2 h, whereas SE-TP_B4_ maintained a stable suspension, confirming enhanced dispersion stability through ball milling.

To further optimize stability, four stabilizers (CMC-Na, sodium alginate, agar, and xanthan gum) were incorporated into tea powder, followed by high-speed homogenization to refine SE-TP_B4_ stability. As shown in Figure 6C, CMC-Na and xanthan gum effectively reduced the centrifugal sedimentation ratio of SE-TP_B4_, while sodium alginate and agar increased it. Increasing stabilizer concentrations universally lowered sedimentation ratios, with low-concentration CMC-Na (0.08 mg/mL) achieving a 92.69% reduction. Though xanthan gum exhibited stabilizing effects, its high viscosity at low concentrations may compromise palatability. Functioning as a hydrocolloid stabilizer, CMC-Na enhanced solution viscosity to inhibit particle aggregation and sedimentation, making it the optimal choice. As shown in Figure 6B, homogenization improved suspension stability by reducing particle size from 4.7 μm to 1.6 μm. Combined treatment with 0.08 mg/mL CMC-Na and homogenization synergistically decreased the centrifugal sedimentation ratio to 9.85% while reducing the median particle size in the supernatant to 1.2 μm. This combination approach enhanced suspension uniformity and stability, demonstrating the complementary effects of stabilizer addition and mechanical processing.

### 3.3. Diffusion Property of Bioactive Compounds of Low-Grade Tea Powder by Steam Explosion and Superfine Grinding

**Figure 7:**
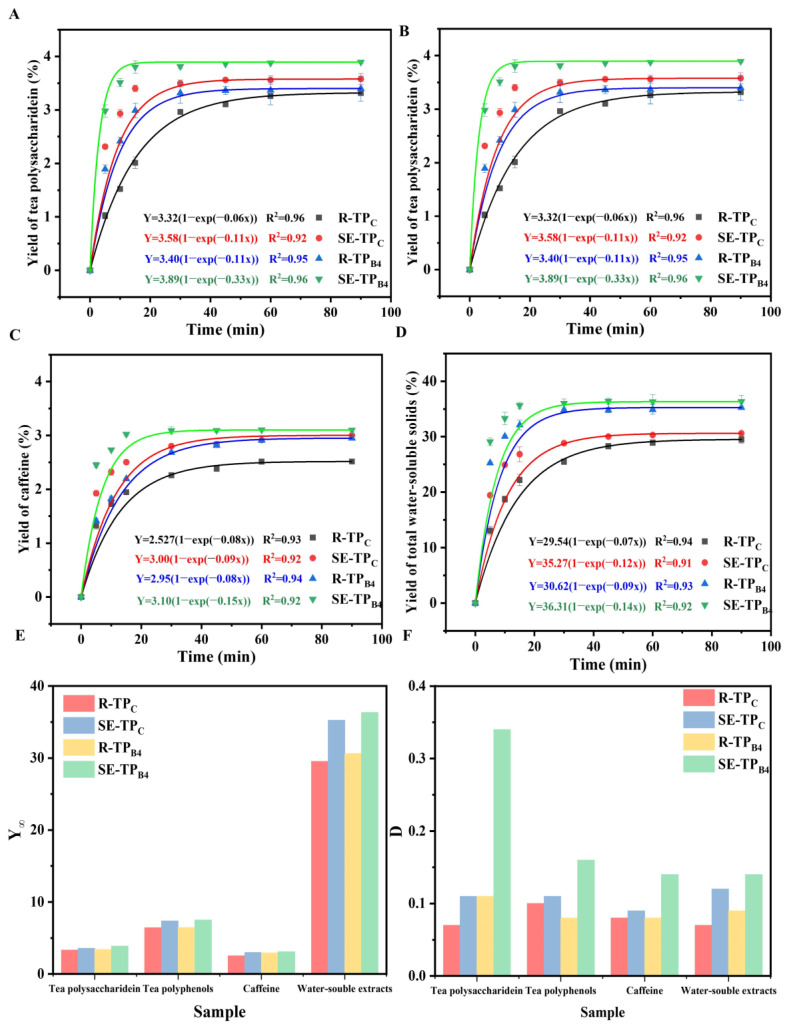
Extraction kinetics of tea polysaccharides, tea polyphenols, caffeine, and total water-soluble solids in tea powder extracts (**A**–**D**); summary of maximum extraction yield *Y*_∞_ and diffusion coefficient *D* from extraction kinetics analysis (**E**,**F**).

To establish optimal extraction parameters for water-soluble components, extraction kinetic studies were systematically performed. Figure 7A–D present the kinetic profiles of active component dissolution in tea infusion, including tea polysaccharides, polyphenols, caffeine, and total water-soluble solids. Fick’s second law was used to model the extraction kinetics, and the fitting parameters of maximum yield *Y*_∞_ and specific rate *D* of bioactive compounds extraction from tea powder were shown in Figure 7E,F. After modeling, Figure 7 shows that the R^2^ values for all fitted equation models exceed 0.9. In previous studies, we adhered to pre-established models, thus eliminating the need for comparison with other models [29]. The fact that the R^2^ values of the fitted curves all exceeded 0.9 sufficiently validates the accuracy and reliability of the model, further justifying the exclusion of alternative models. We used the prior model framework and methodology to maintain consistency and efficiency in our analysis. SE samples demonstrated superior extraction performance compared to untreated counterparts, achieving peak bioactive compound recovery within significantly reduced processing durations. For all tea powder, the bioactive extract yield increased with the extraction time, and once a certain time was reached, the yield varied little, indicating that the extraction reached equilibrium [30].

As shown in Figure 7A, the equilibrium time for tea polysaccharides in R-TP_C_ and SE-TP_C_ was 60 min and 30 min, respectively. The *Y*_∞_ values for tea polysaccharides were 3.32% for R-TP_C_ and 3.58% for SE-TP_C_, with the latter being 1.08 times higher than the former. Comparing R-TP_B4_ and SE-TP_B4_, their equilibrium times were approximately 30 min and 15 min, respectively. The *Y*_∞_ values for tea polysaccharides were 3.40% for R-TP_B4_ and 3.89% for SE-TP_B4_, with the latter being 1.14 times higher than the former. The *D* increased under all four conditions, indicating that partial hydrolysis of cell wall components and hydrothermal decomposition of the porous matrix significantly improved solute-solvent accessibility, facilitating the dissolution and release of active components. Comparing SE-TP_C_ and SE-TP_B4_, the extraction yield of tea polysaccharides for SE-TP_B4_ significantly increased to 3.80% after 15 min, whereas it was 3.40% for SE-TP_C_ under the same times, making the former 1.12 times higher. The *Y*_∞_ and *D* values for SE-TP_B4_ were 1.09 and 3.09 times those of SE-TP_C_, respectively. This demonstrates that SE-ball milling significantly enhances the extraction yield of tea polysaccharides.

As shown in Figure 7B, the equilibrium times for tea polyphenols in R-TP_C_ and SE-TP_C_ were 60 min and 45 min, respectively. The *Y*_∞_ values for tea polyphenols were 6.42% for R-TP_C_ and 6.46% for SE-TP_C_, with the latter being 1.01 times higher. Comparing R-TP_B4_ and SE-TP_B4_, their equilibrium times were approximately 45 min and 30 min, respectively. The *Y*_∞_ values for tea polyphenols were 6.46% for R-TP_B4_ and 7.50% for SE-TP_B4_, with the latter being 1.16 times higher. The *D* increased under all four conditions, indicating that SE significantly enhances the extraction yield of tea polyphenols. Comparing SE-TP_C_ and SE-TP_B4_, the extraction yield of tea polyphenols for SE-TP_B4_ significantly increased to 7.51% after 30 min, whereas it was 7.22% for SE-TP_C_ under the same times, making the former 1.04 times higher. The *Y*_∞_ value for SE-TP_B4_ was 1.02 times that of SE-TP_C_. The findings indicate that reducing the particle size of tea powder through SE-ball milling coupling treatment enhances tea polyphenol dissolution [31]. Consistent with these results, Qiu et al. demonstrated that effective cell wall disruption and the high specific surface area of smaller particles accelerate the dissolution rate of active components, thereby reducing the time required for these components to diffuse through the particle matrix. The equilibrium dissolution time of tea polyphenols significantly decreases with smaller particle sizes, being 4 times faster than in raw tea powder [32,33]. All four treatment methods enhanced the maximum extraction yield of polyphenols. However, common polyphenols typically have melting points above 200 °C, and may begin to degrade or undergo structural changes at temperatures exceeding their melting points. Contrary to expectations, our experimental data showed no decline in the content of these bioactive substances during the extraction process combining SE and ultrafine grinding technologies. The reasons for this are primarily twofold: first, the SE treatment duration is brief, insufficient to trigger significant degradation reactions; second, although SE does induce thermal degradation, its effect in promoting the extraction of target compounds surpasses the degradation effect, resulting in an overall increase in extraction yield. In subsequent research, we will further investigate the specific changes of polyphenolic compounds during the SE process.

As shown in Figure 7C, the equilibrium times for caffeine in R-TP_C_ and SE-TP_C_ were 60 min and 45 min, respectively. The *Y*_∞_ values for caffeine were 2.52% for R-TP_C_ and 3.00% for SE-TP_C_, with the latter being 1.19 times higher. Comparing R-TP_B4_ and SE-TP_B4_, their equilibrium times were approximately 45 min and 30 min, respectively. The *Y*_∞_ values for caffeine were 2.95% for R-TP_B4_ and 3.10% for SE-TP_B4_, with the latter being 1.05 times higher. Therefore, SE significantly enhances the extraction yield of caffeine. Comparing SE-TP_C_ and SE-TP_B4_, the extraction yield of caffeine for SE-TP_B4_ significantly increased to 3.09% after 30 min, whereas it was 2.80% for SE-TP_C_ under the same times, making the former 1.10 times higher. The *Y*_∞_ and *D* values for SE-TP_B4_ were 1.03 and 1.56 times those of SE-TP_C_, respectively, which is three times faster than that for SE-TP_C_ treatment. This suggests that SE-TP_B2_ significantly disrupted cell wall structure, reduced mass transfer resistance of active components in the solvent, and enhanced the diffusion rate of caffeine in water [34]. However, caffeine has a melting point of approximately 178 °C. At or above this temperature, caffeine undergoes dehydration and ring cleavage reactions, forming various compounds and thus reducing its content. Interestingly, the anticipated decline in caffeine levels was not observed in this study. We hypothesize that the rapid temperature fluctuations and brief contact time during the extraction process effectively minimized thermal degradation.

As shown in Figure 7D, the equilibrium times for water-soluble solids in R-TP_C_ and SE-TP_C_ were 90 min and 60 min, respectively. The *Y*_∞_ values for water-soluble solids were 29.54% for R-TP_C_ and 35.27% for SE-TP_C_, with the latter being 1.19 times higher. Comparing R-TP_B4_ and SE-TP_B4_, their equilibrium times were approximately 45 min and 30 min, respectively. The *Y*_∞_ values for water-soluble solids were 30.62% for R-TP_B4_ and 36.32% for SE-TP_B4_, with the latter being 1.19 times higher. Therefore, SE significantly enhances the extraction yield of water-soluble solids. Comparing SE-TP_C_ and SE-TP_B4_, the extraction yield of water-soluble solids for SE-TP_B4_ significantly increased to 36.02% after 30 min, whereas it was 28.82% for SE-TP_C_ under the same times, making the former 1.25 times higher. The *Y*_∞_ and *D* values for SE-TP_B4_ were 1.03 and 1.17 times those of SE-TP_C_, respectively.

Overall, when extracting active compounds from tea powder using SE treatment alone, the technique significantly increases the specific surface area of tea powder from a physical perspective. This enhances the permeability of the extraction solvent through the cell walls, facilitating the release of polysaccharides, polyphenols, and caffeine from the intercellular spaces. Chemically, the high-temperature and high-pressure hydrothermal environment generated by SE promotes the cleavage of glycosidic bonds in polysaccharides, producing smaller, more easily extractable molecules [35]. SE also breaks ester and hydrogen bonds in lignin-polysaccharide complexes, weakening the binding force between tea polyphenols and cell wall components [36]. Additionally, the high temperature may induce the hydrolysis of polyphenol ester bonds, generating smaller hydrophilic derivatives. For caffeine, SE might convert its bound form with macromolecules such as proteins into a free form. The increase in water-soluble substances is primarily due to the high temperature promoting chemical reactions that generate new water-soluble compounds [37]. For its physiological activity, polysaccharides treated with SE usually exhibit reduced molecular weight and increased solubility, thereby enhancing their antioxidant, immunomodulatory, and antitumor activities [35].

Therefore, when considering the dissolution amount and dissolution equilibrium time of bioactive compounds. The order of treatment methods that yield the best extraction of active substances is SE-TP_B4_ > SE-TP_C_ > R-TP_B4_ > R-TP_C_. This could be attributed to the fact that SE and ball milling further enhance the accelerated dissolution of bioactive compounds. In summary, SEM observations in Section 3.2.1 revealed that the loose and porous structure on the surface of tea powder treated by steam SE significantly enhanced the grinding efficiency in subsequent milling processes. The particle size distribution analysis in Section 3.2.2 indicated that SE-TP_B4_ achieved the smallest *D*_50_ of 10.4 ± 0.17 μm, with a cell wall breakage ratio of 99.9% under these conditions. This suggests that SE-TP_B4_ improved solute-solvent accessibility, facilitating the extraction of more components by penetrating the cell walls.

### 3.4. Industrial Scalability and Energy Consumption Analysis

The industrial feasibility of SE technology for processing low-grade tea residues was evaluated through a comprehensive analysis of equipment scalability, energy efficiency, and process economics. SE systems have been successfully scaled to industrial volumes (50 m^3^ reactors) for lignocellulosic biomass processing, demonstrating robustness in handling large feedstock quantities [35,38,39].

**Figure 8:**
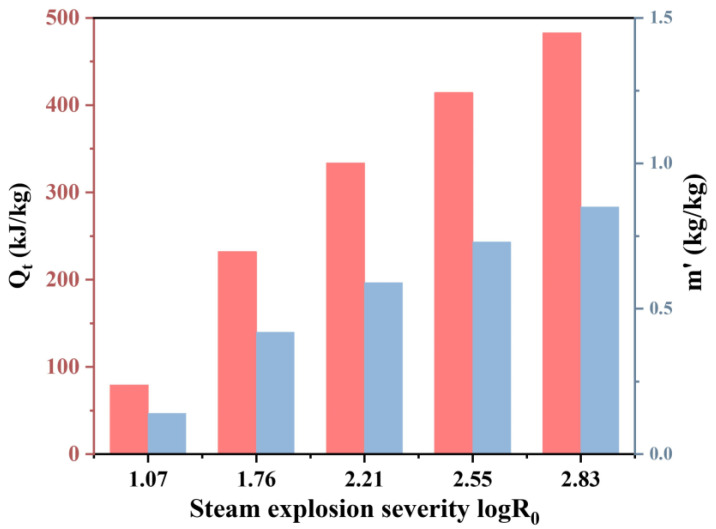
Total energy consumption (*Q_t_*) and unit dry matter steam consumption (*m^′^*) under different SE severities.

Energy expenditure was systematically quantified across five SE severities, revealing a nonlinear correlation between process intensity and energy demand. As shown in Figure 8, total energy consumption (*Q_t_*) and unit dry matter steam consumption (*m^′^*) escalated significantly with increasing severity. At the lowest severity (1.07 log*R*_0_), *Q_t_* and m^′^ were 79.16 kJ/kg and 0.14 kg/kg, respectively. Under the highest severity (2.83 log*R*_0_), these values surged to 482.62 kJ/kg and 0.85 kg/kg, underscoring the inherent trade-off between bioactive compound diffusivity and energy efficiency. A multivariate regression model (*m^′^* = 0.77 + 0.051*B* + 0.38*C* − 0.48*D* + 1.28*E* − 0.034*BD* − 0.13*CD* + 0.18*CE* + 0.57*D*^2^ + 0.82*E*^2^) identified moisture content (*C*) and holding time (*E*) as pivotal determinants of steam efficiency.

While high-severity SE (log*R*_0_ = 2.83) maximizes the solvent accessibility of bioactive compounds, its annual energy cost presents a critical barrier to industrial adoption. Transitioning to continuous SE systems could mitigate thermal cycling losses and enhance scalability, yet the substantial capital investment required for such infrastructure remains prohibitive for small-scale operations. Future research should prioritize pilot-scale validation of these models under industrial conditions, coupled with lifecycle assessments to quantify environmental footprints. By optimizing process parameters and modular reactor designs, SE technology offers a sustainable framework to valorize low-grade tea into high-value functional ingredients, aligning with circular economy principles.

## 4. Conclusions

This study demonstrated the efficacy of steam explosion combined with superfine grinding in enhancing the instant solubility and diffusivity of low-grade tea powder. SE treatment induced critical structural and chemical modifications, including partial hemicellulose hydrolysis, lignin recondensation, and cellulose crystalline region reorganization. These alterations weakened the lignocellulosic matrix, as evidenced by SEM observations of porous architectures and disrupted vascular bundles, thereby facilitating subsequent mechanical comminution. Ball milling synergized with SE pretreatment to achieve ultrafine particle refinement, with SE-TP_B4_ (steam-exploded, ball-milled 4 h) exhibiting a median particle size (*D*_50_) of 10.4 ± 0.17 μm and near-complete cell disruption (99.9%). Comparative analysis revealed that ball milling outperformed cryogenic grinding in particle homogeneity (span factor reduced to 2.1) due to its dynamic shear forces targeting SE-weakened amorphous regions. The sedimentation property of SE-TPB_4_ was further improved by homogenization and combination with 0.08 mg/mL CMC-Na, which achieved the minimal centrifugal sedimentation (9.85%) and uniform suspension stability (*D*_50_ = 1.2 μm).

Extraction kinetics analysis underscored the superiority of SE-ball milling in accelerating the diffusion process of bioactive compounds. SE-TP_B4_ reached the extraction equilibrium time 2~4 times faster than untreated samples, with maximum yields (*Y*_∞_) of tea polysaccharides (3.89%), polyphenols (7.50%), caffeine (3.10%), and water-soluble solids (36.32%), surpassing SE-TP_C_ by 9~25%. These enhancements were attributed to the near-total cell wall breakage ratio, which reduced mass transfer resistance and improved solute-solvent accessibility. The findings align with previous studies emphasizing the role of particle size reduction and structural disintegration in optimizing the bioavailability of natural products. Additionally, functional teas also contain abundant amino acids, volatile compounds, and specific flavonoids. Studies have shown that steam explosion treatment significantly promotes the extraction and release of these substances, thereby enhancing their content and composition. In future research, we will explore the interactions among these various nutrients and their combined effects on health further, aiming to gain a comprehensive understanding of the health benefits of functional tea.

The SE treatment demonstrates significant advantages in enhancing bioactive compound extraction and structural modification of agricultural biomass, achieving near-complete cell wall breakage ratios and superior extraction yields of bioactive compounds compared to traditional methods. For instance, SE treatment of rice straw attained 70% cellulose extraction efficiency with effective lignin recovery while markedly improving fiber porosity and surface area [40]. Similarly, when applied to cereal processing by-products, this technology increased soluble dietary fiber content by 171%, transforming dietary fibers into honeycomb-like porous structures that enhance hydration capacity, glucose-lowering effects, and antioxidant activity [41]. These structural and functional improvements position SE combined with superfine grinding as a promising solution for upgrading low-grade tea powder, addressing challenges such as sedimentation stability, dispersion homogeneity, and bioactive compound diffusivity in instant beverage applications. However, industrial scalability faces hurdles, including high energy demands during SE processing and potential sensory alterations, necessitating further pilot-scale trials to optimize energy efficiency and develop synergistic strategies for product refinement. This integrated approach not only adds value to underutilized agricultural byproducts like low-grade tea but also advances sustainable resource utilization, establishing a framework for converting waste materials into high-performance functional ingredients for food and pharmaceutical industries.

## Data Availability

The original contributions presented in this study are included in the article. Further inquiries can be directed to the corresponding author.
